# Effect of the decreased frequency of going out on the association between anxiety and sleep disorder during the COVID-19 pandemic: a mediation analysis

**DOI:** 10.1186/s12991-023-00456-z

**Published:** 2023-07-23

**Authors:** Yumi Sugawara, Yutaka Yabe, Yoshihiro Hagiwara, Ichiro Tsuji

**Affiliations:** 1 Division of Epidemiology, Department of Health Informatics and Public Health, Graduate School of Medicine, Tohoku University School of Public Health, 2-1, Seiryo-Machi, Aoba-Ku, Sendai, Miyagi 980-8575 Japan; 2 Department of Orthopaedic Surgery, Tohoku University School of Medicine, Sendai, Japan

**Keywords:** COVID-19 pandemic, Anxiety, Sleep disorder, Frequency of going out, Mediation analysis

## Abstract

**Purpose:**

The objective of the present study was to examine the relationship between anxiety and sleep disorder during the COVID-19 pandemic and to evaluate whether sleep disorder is mediated by the decreased frequency of going out.

**Methods:**

The data of a total of 1976 residents aged 18 years and over who had responded to a self-reported questionnaires at a health survey in 2020 were analyzed. The subjects were divided into four groups based on their response to the questionnaire on anxiety about the COVID-19 pandemic. Sleep disorder was measured using the Athens Insomnia Scale (AIS). A cross-sectional analysis was performed to examine the association between anxiety about the COVID-19 pandemic and AIS scores. Mediation analysis was used to calculate the association between anxiety and AIS scores during the COVID-19 pandemic, with decreased frequency of going out as a potential mediating variable.

**Results:**

In the cross-sectional study, the level of anxiety about the COVID-19 pandemic was significantly associated with the AIS score (*p* < 0.001). On mediation analysis, the direct effect of the relationship showed that anxiety positively influenced AIS scores (β = 0.283, *p* < 0.01). The indirect effect of the relationship showed that the decreased frequency of going out positively mediated the relationship between anxiety and AIS scores (β = 0.342, *p* < 0.05). The decreased frequency of going out accounted for almost 10% of the AIS score.

**Conclusion:**

The present study found that anxiety about the COVID-19 pandemic was significantly associated with sleep disorder, with the decreased frequency of going out mediating this association.

## Introduction

The COVID-19 pandemic of 2019–2022 is having serious effects worldwide, with a large impact on mental health [1, 2]. Sleep disorders like insomnia are common mental health problems during the COVID-19 pandemic and may be important predictors of other mental health problems, such as depression, alcohol abuse, drug abuse, and suicide [3, 4]. Sleep can play an important role in regulating the immune function and host defense in the outbreak of COVID-19 [5]. Previous studies suggest that sleep affects various immune parameters, is associated with a reduced infection risk, and can improve infection outcome and vaccination responses [6, 7]. Meanwhile, several studies have shown that anxiety is strongly associated with sleep disorders during the COVID-19 pandemic [8, 9]. The results of a web-based survey of Portuguese adults reported that participants who reported lower levels of anxiety had more satisfaction with sleep quality [8]. A study conducted in Italy also reported that a higher level of anxiety predicted poorer sleep quality and more severe insomnia symptoms [9]. However, it is not clear which factors might explain the association between anxiety and sleep disorders during the pandemic.

The decreased frequency of going out might be related to both anxiety and sleep disorders during pandemic. Some previous studies showed that the decreased frequency of going out was associated with anxiety [10, 11]. Data from the COVID-19 Social Study in the UK showed that the change in days spent outside was negatively associated with the change in anxiety symptoms [10]. Similarly, a web-based, cross-sectional study in Austria reported that, compared with participants who spent < 60 min/day outdoors, participants who spent ≥ 60 min/day outdoors had fewer anxiety symptoms (OR = 0.70; 95%CI 0.49–1.02) [11]. On the other hand, data from several studies suggest that time spent outside the home was significantly associated with sleep quality during the pandemic [12–14]. For example, a cross-sectional study conducted in Italy has shown that poor sleep quality was predicted by a greater lockdown-related increase of the time spent at home [14]. However, the impact of decreased outings on the association between anxiety and sleep disorder during the pandemic remains unknown.

In Japan, there was no European-style hard lockdown for prevention of the spread of COVID-19. The Japanese government urged people to work from home if possible and to only go out when necessary as a means of minimizing the risk of infection [15]. Because it was not a hard enforced lockdown, peoples’ reactions varied widely, and those with high anxiety about COVID-19 tended to stay home. A cross-sectional study in Japan reported that anxiety regarding going outside was significantly associated with a decreased frequency of going out among community-dwelling older adults after the beginning of the pandemic [16]. Thus, there is a possibility that the effect of refraining from going out on health might vary in view of the differences in the basic response policy for preventing the spread of COVID-19 between Japan and other countries. However, little is known about the impact of the decreased frequency of going out due to the COVID-19 pandemic on sleep disorders in the Japanese population.

The objective of the present study was to examine the relationship between anxiety and sleep disorder during the COVID-19 pandemic and to evaluate whether the presence of sleep disorder is mediated by a decreased frequency of going out.

## Methods

### Study design and participants

This study was based on the health survey of the Great East Japan Earthquake (GEJE) survivors conducted every year since the GEJE in Miyagi Prefecture, Japan. The design of the health survey of the GEJE survivors has been described in detail elsewhere [17–20]. In brief, between July and August 2020, a self-reported questionnaire was administered to residents aged 18 years or older who were living in Ogatsu and Oshika, located in Ishinomaki City, which suffered extensive damage in the GEJE in Miyagi Prefecture, northeastern Japan (the health survey in 2020). The questionnaire included items on residents’ life environment, such as housing type, medical history, self-rated health, economic status, smoking habits, drinking habits, dietary habits, sleep disorder (based on the 8-item Athens Insomnia Scale [AIS]) [21], psychological distress (based on the 6-item Kessler Psychological Distress Scale [K6]) [22], working status, and social network (based on the 6-item Lubben Social Network Scale [LSNS-6]) [23, 24]. Three items about the effect of the COVID -19 pandemic (anxiety, frequency of going out, and income) were added to the health survey in 2020.

**Fig. 1:**
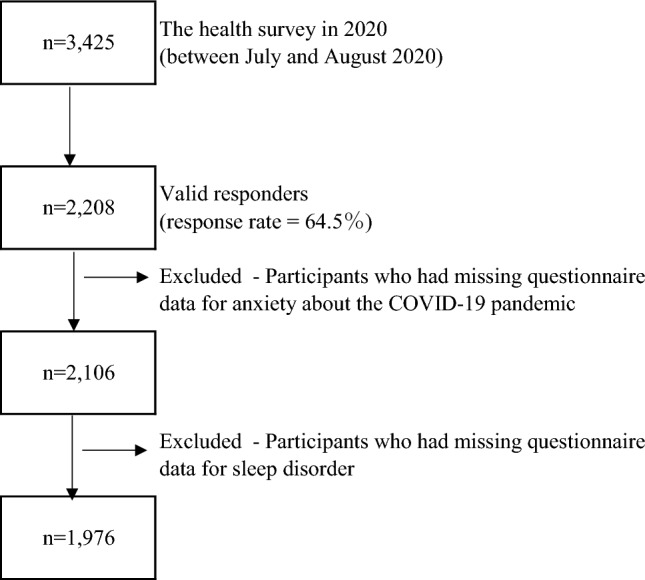
Flow diagram

Of the 3,425 eligible subjects, 2,208 responded to the health survey in 2020 (response rate: 64.5%); 102 subjects who had missing questionnaire data for anxiety about the COVID-19 pandemic were excluded. Another 30 subjects who had missing questionnaire data for sleep disorders were also excluded. Thus, 1,976 subjects were included in the study cohort (Fig. 1).

### Exposure variable: anxiety about the COVID-19 pandemic

Anxiety about the COVID-19 pandemic was assessed by responses to the following question: “How often do you feel anxious about the COVID-19 pandemic?”. The subjects were divided into four groups based on their responses to the question: “never”, “a little bit”, “sometimes”, and “usually or always”.

### Outcome variable: sleep disorder

Sleep disorder was measured using the Athens Insomnia Scale (AIS) [21]. The AIS is an individual’s own assessment of any sleep disorder he/she might have experienced, provided that it occurred at least three times per week during the last month. It contains eight questions rated from 0 to 3, with a total score ranging from 0 to 24. A higher total AIS score indicates poorer of sleep status, and 6 or above indicates a sleep disorder [21].

### Mediating variable: the decreased frequency of going out during the COVID-19 pandemic

The decreased frequency of going out during the COVID-19 pandemic was assessed by responses to the following question: “Did you change the frequency of going out due to the COVID-19 pandemic?”. The subjects were divided into four groups based on their responses to the question: “never changed”, “reduced by 20–30%”, “reduced by half”, and “hardly ever went out”.

### Covariates

Self-rated health was evaluated by asking about health status (very good, good, poor, or bad). Self-rated health was categorized into two groups: “very good” and “good” or “poor” and “bad”. Economic status was evaluated by asking about current household economic status as assessed by degree of financial difficulty (very difficult, difficult, a little difficult, or normal). Economic status was categorized into two groups: “very difficult” and “difficult” or “a little difficult” and “normal”.

### Statistical analysis

First, the characteristics of the study subjects at the health survey in 2020 were examined. Then, the mean AIS scores at the health survey in 2020 were examined across the anxiety groups. ANOVA was used to analyze the association between anxiety and mean AIS scores. Third, mediation analyses were performed for the association between anxiety and sleep disorder during the COVID-19 pandemic, with decreased frequency of going out as a potential mediating variable. Stata’s causal mediation analysis was used to estimate the standardized total, direct, and indirect effects and the proportion of the total effect mediated by the decreased frequency of going out after adjusting for covariates. Standard errors and confidence intervals were obtained using the bootstrapping method (1000 repetitions) [25].

The following variables were considered possible confounders: age (continuous variable), sex (male or female), self-rated health [good (very good or good), poor (poor or bad), or missing], and economic status [severe (very difficult or difficult), normal (a little difficult or normal), or missing]. These items were chosen as covariates because both health and economic status may affect the association between anxiety and sleep disorders.

All *P* values were two-sided, and differences at *p* < 0.05 were considered significant. Mediation analyses were conducted using Stata Statistical Software, version 16.0 (Stata Corp LLC, College Station, TX, USA). All other statistical analyses were performed using the SAS statistical software package, version 9.4 (SAS Institute Inc, Cary, NC, USA).

### Ethical issues

The research related to human use has been complied with all the relevant national regulations, institutional policies and in accordance the tenets of the Helsinki Declaration. The study protocol was approved by the Institutional Review Board of Tohoku University Graduate School of Medicine (approved number: 2011–92, 2020–1-465). Consent to participate in the study was obtained from the participants either face-to-face or through the signed self-administered questionnaire.

## Results

### Participants’ characteristics

Table 1 shows the characteristics of the study participants at the health survey in 2020. There were 897 male participants and 1,079 female participants. In the health survey in 2020, 435 (22.0%) participants had poor self-rated health, and 216 (10.9%) of them had psychological distress (K6 score, ≥ 10 points). Furthermore, for the effect of the COVID-19 pandemic, 1029 (52.1%) participants reported usually/always had anxiety, 414 (21.0%) participants reported almost hardly ever went out, and 136 (6.9%) participants reported income reduced by more than 70%.

**Table 1:** Characteristics of the 1976 participants at health survey in 2020

Participants	All	
	1976	
Sex (%)		
Men	897	(45.4%)
Women	1079	(54.6%)
Age, years (mean ± SD)	62.3 (19.5)	
Housing type (%)		
Same as before the GEJE	544	(27.5%)
Privately rented temporary/rental	109	(5.5%)
Reconstructed	670	(33.9%)
Disaster public housing^a^	499	(25.3%)
Others	123	(6.2%)
Self-rated health (%)		
Good (very good/good)	1515	(76.7%)
Poor (poor/bad)	435	(22.0%)
Job status (%)		
Employed	997	(50.5%)
Unemployed	954	(48.3%)
Economic status (%)		
Normal (a little difficult/normal)	1011	(51.2%)
Severe (very difficult/difficult)	960	(48.6%)
Drinking status (%)		
Currently drinking	606	(30.7%)
Non-drinking	1273	(64.4%)
Time spent walking (%)		
≥ 1 h/day	580	(29.4%)
0.5–1.0 h/day	710	(35.9%)
< 0.5 h/day	665	(33.7%)
Psychological distress^c^ (%)		
< 10	1673	(84.7%)
≥ 10	216	(10.9%)
Social network^d^ (%)		
< 12	613	(31.0%)
≥ 12	1362	(68.9%)
Effect of the COVID-19 pandemic		
Anxiety		
Never	58	(2.9%)
a lltle bit	397	(20.1%)
Sometimes	492	(24.9%)
Usually/always	1029	(52.1%)
Frequency of going out		
Not changed	380	(19.2%)
Reduced by 20–30%	566	(28.6%)
Reduced by half	608	(30.8%)
Hardly ever went out	414	(21.0%)
Income		
Not changed	1266	(64.1%)
Reduced by 20–30%	320	(16.2%)
Reduced by half	171	(8.7%)
Reduced by 70% over	136	(6.9%)

^a^ Disaster public housing; recovery public housing or collective relocation for disaster prevention

^b^ Athens Insomnia scale score

^c^ Kessler 6-item psychological distress scale score

^d^ Lubben social network scale-6 score

*SD* standard deviation

### Anxiety and sleep disorder (AIS scores)

Table 2 shows the mean AIS scores at the health survey in 2020 according to anxiety group. The mean AIS scores ± standard deviations at the health survey in 2020 were 2.60 ± 3.66 for participants who never had anxiety, 3.44 ± 3.03 for those who had a little bit of anxiety, 4.17 ± 3.08 for those who sometimes had anxiety, and 5.23 ± 3.97 for those who usually/always had anxiety. The level of anxiety about the COVID-19 pandemic was significantly associated with the AIS score at the health survey in 2020 (*p* < 0.001).

**Table 2:** The mean AIS scores at the health survey in 2020 according to anxiety about the COVID-19 pandemic

	Anxiety				
	Never	A lltle bit	Sometimes	Usually/always	*p* value
	(n = 57)	(n = 397)	(n = 492)	(n = 1029)	
AIS score (mean ± SD)	2.60 ± 3.66	3.44 ± 3.03	4.17 ± 3.08	5.23 ± 3.97	< 0.001

*SD* standard deviation

### Mediation analysis: *effects of the decreased frequency of going out*

**Fig. 2:**
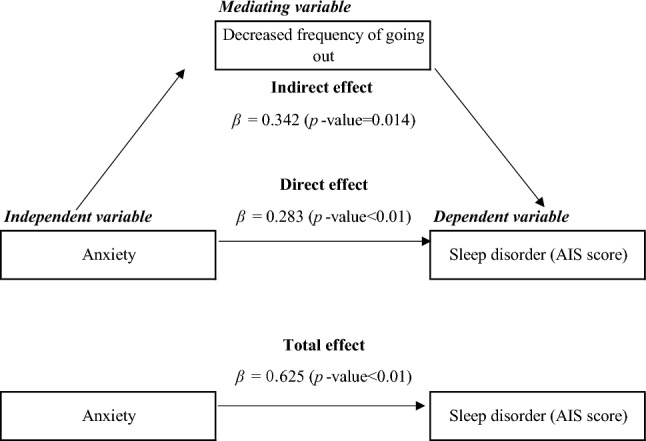
The mediating role of decreased frequency of going out in the association between anxiety and sleep disorder during the COVID-19 pandemic

Table 3 shows the mediation analysis for the association between anxiety and sleep disorder during the COVID-19 pandemic. The adjusted mediation analysis showed that the association between anxiety and sleep disorder was partially mediated by the decreased frequency of going out during the COVID-19 pandemic. The direct effect of the relationship showed that anxiety positively affected AIS scores (*β* = 0.283, *p* < 0.01). The indirect effect of the relationship showed that the decreased frequency of going out positively mediated the relationship between anxiety and AIS scores (*β* = 0.342, *p* < 0.05). The AIS scores during the COVID-19 pandemic were explained by almost 10% by the decreased frequency of going out. The summary of the mediation analysis is presented in Fig. 2.

**Table 3:** Mediation effect of the decreased frequency of going out on the association between anxiety and sleep disorder (AIS score)

	Indirect effect		Direct effect		Total effect		Proportion of total effect mediated
	Estimate	95%CI^a^	Estimate	95%CI^a^	Estimate	95%CI^a^	%
Decreased frequency of going out	0.342*	− 0.118, 0.802	0.283**	0.081, 0.485	0.625**	0.313, 0.936	9.9

^*^p < 0.05, **p < 0.01

^a^ Adjusted for age (continuous variable), sex (male or female), self-rated health (good, poor, or missing), and economic status (severe, normal, or missing)

## Discussion

In the cross-sectional study of 1,976 GEJE survivors, nearly 50% of participants felt anxiety about the COVID-19 pandemic. Anxiety about COVID-19 was found to be associated with sleep disorder in natural disaster survivors. Moreover, the present study showed that the association between anxiety about the COVID-19 pandemic and sleep disorder was partially mediated by the decreased frequency of going out. To the best of our knowledge, this is the first study to investigate the mediation effect of the decreased frequency of going out on the association between anxiety and sleep disorder.

### Relationship between anxiety and sleep disorder

The results of the present study showed that severe anxiety about the COVID-19 pandemic was significantly associated with poor sleep status. It is well known that anxiety might negatively affect sleep status [26, 27]. Several COVID-19 studies have also reported the association between anxiety and sleep disorder [8, 9]. The present result is consistent with those previous studies.

### Effects of decreased frequency of going out on the association between anxiety and sleep disorder

Mediation modeling was used to examine whether sleep status is mediated by changes in the frequency of going out. Going out may particularly relieve stress from anxiety during the pandemic. Some cross-sectional studies conducted during the pandemic have shown that a decreased frequency of going out was positively associated with anxiety [10, 11]. In addition, previous studies have shown that spending time outside was associated with mental and physical health [28, 29]. One study showed that even just 20 min per day spent in nature can lower stress hormone levels, boost self-esteem, and improve mood [28]. Data from a large nationally representative sample in the UK showed that people who spent two hours a week or more outdoors reported being in better health and having a greater sense of well-being than people who did not get out at all [29]. The present study supports the results of these previous studies.

The indirect effect of the decreased frequency of going out on sleep disorder in the present participants was found to be 9.9%, lower than that of previously reported levels. The Evolution of Pathways to Insomnia Cohort (EPIC) study examined the association between stress exposure and the development of insomnia, and they reported that the effect of stress exposure on risk for insomnia was mediated by three specific coping behaviors (behavioral disengagement, distraction, and substance use) [29]. The proportion of partial mediation in the total effect was 21% for substance use, 86% for self-distraction, and 91% for behavioral disengagement. Therefore, the decreased frequency of going out could be one factor, if not a major factor, mediating an increased risk of sleep disorder by anxiety.

The frequency of going out connects us more with the people and places in our community. Recent evidence suggests that loneliness and lack of a social network were associated with poorer sleep [30]. For example, Cacioppo et al. reported that increased loneliness leads to reduced sleep quality [30]. Another study reported that both women and men with low social support had poorer sleep quality [31]. Thus, a reduced frequency of going out might also lead to attrition of social networks and social support, leading to poorer sleep quality.

To minimize social and economic impacts, Japan’s government did not enforce compulsory measures such as lockdowns that have been implemented in other countries, but rather called on the population for a calm response (self-restraint from travel across prefectures, such as for nonessential visits to hometowns and leisure travel, avoiding the flooding of shops and panic buying) [15]. However, the present study showed that the relationship between anxiety and sleep disorder during the COVID-19 pandemic was mediated by a decreased frequency of going out. This finding provided evidence for the impact of decreased outings on sleep disorder in the Japanese population who had not experienced a hard lockdown. Further research conduct in the same sample should evaluate the impact of decreased outings due to the COVID-19 pandemic on mental health. In addition, there are needed to evaluate whether the change of frequency of going out during the COVID-19 pandemic contribute to reduction on mental health.

## Limitation

The present study had several limitations. First, it was a cross-sectional study, and future studies are needed to explore the causal connection in a longitudinal study design. Second, most data collected from the study participants were based on the self-reported questionnaire. Thus, some non-differential misclassification would have been inevitable. Third, the participants of this study were GEJE survivors, and they might have more severe anxiety and chronic sleep disorders (e.g. insomnia, circadian sleep–wake rhythm disturbances) than the general population. However, the prevalence of sleep disorder (AIS score ≥ 6) at the 2020 health survey in the present participants was broadly consistent with those of a previous study (15.2% vs. 17.4%) [32]. Fourth, no information about history of mental illness and medication, which could also be associated with the risk of sleep disorders, was collected. Therefore, the results may underestimate the mediating effect by the decreased frequency of going out on the relationship between anxiety and sleep disorder during the COVID-19 pandemic. Finally, information only about the decreased frequency of going out was collected; therefore, it was not possible to investigate the association between types of outdoor activities or hours spent outdoors and sleep disorder.

## Conclusion

The present study showed that anxiety about the COVID-19 pandemic was significantly associated with sleep disorder, with the decreased frequency of going out mediating this association.

## Data Availability

Data presented in this study are available on request from the corresponding author on a reasonable request.
